# Supplementary material for: The Cell Polarity Protein MPP5/PALS1 Controls the Subcellular Localization of the Oncogenes YAP and TAZ in Liver Cancer
Source: Int J Mol Sci. 2025 Jan 14;26(2):660. doi: 10.3390/ijms26020660 (PMC11766031; doi:10.3390/ijms26020660)
Supplement: Supplementary file 1 [file ijms-26-00660-s001.zip › ijms-3385530-supplementary.pdf]

International Journal of
Molecular Sciences

MDPI

Supplementary Information

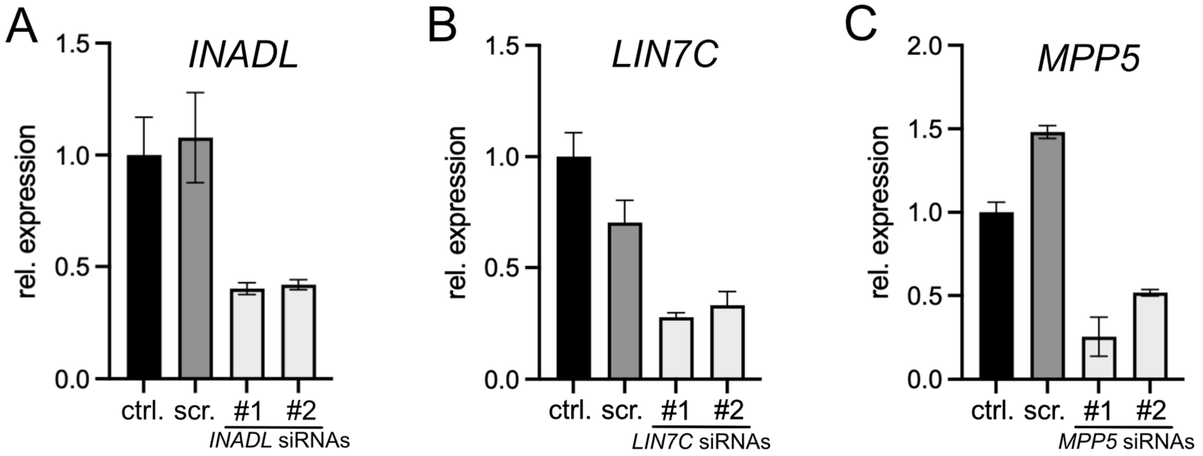

Figure S1. Exemplary confirmation of FlexiPlate target genes. Real-time PCR analysis of **(A)** *INADL*, **(B)** *LIN7C*, and **(C)** *MPP5* after transfection of the respective siRNAs in HepG2 cells. Untreated cells (ctrl.) and scrambled siRNA (scr.)-transfected cells were used as controls.

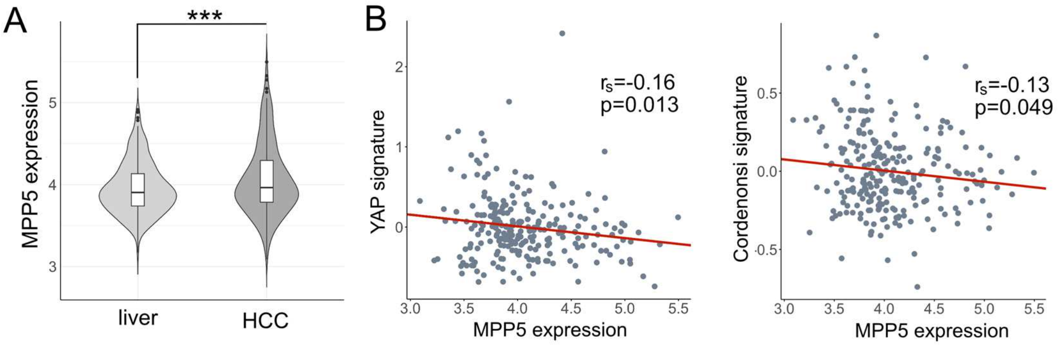

Figure S2. *MPP5* negatively correlates YAP target gene expression and clinical outcome in HCC patients (independent cohort). **(A)** Transcriptome analysis of *MPP5* mRNA levels in HCC tissues compared to surrounding liver tissues. Mann–Whitney U test. **(B)** Association between MPP5 and YAP target gene signature expression (Wang and Cordenonsi signatures). Spearman correlation analysis.

Table S1. FlexiPlate siRNAs.

Gene name	Gene ID	Target sequence
AMOT #2	154796	CACCAACGTTTCAGAATACAA
AMOT #6	154796	ATGCGGCAGCTTAAAGTGTAT
AMOTL1 #5	154810	CAACGAGGAACTGCCCACTTA
AMOTL1 #6	154810	AACTCCTAGAATCACCTATAA
AMOTL2 #5	51421	CTGTATGTTTAAGTTATCGTA
AMOTL2 #6	51421	AAACTAGTTAATGAGCTACAA
ARHGAP17 #3	55114	CCCAAGCAGATTACCATAGAA
ARHGAP17 #5	55114	AAGCAGTGCGTTAACTATCTA

CDC42 #4	998	TTCAGCAATGCAGACAATTAA
CDC42 #7	998	CATCAGATTTGAAATATTTAA
CDH1 #12	999	CTAGGTATTGTCTACTCTGAA
CDH1 #13	999	TCGGCCTGAAGTGACTCGTAA
CRB3 #2	92359	CCAGTGCTTAATAGCAGGGAA
CRB3 #6	92359	TTAGTAGTGAGATGTAACAAA
CTNNA1 #6	1495	AAGTGGATAAGCTGAACATTA
CTNNA1 #8	1495	GCGAATTGTGGCAGAGTGTAA
CTNNB1 #5	1499	CTCGGGATGTTCACAACCGAA
CTNNB1 #9	1499	CAGGATGATCCTAGCTATCGT
DLG3 #5	1741	ACGGCTTGTGAAGTGAGCTAA
DLG3 #6	1741	AGGCGGGCCAATTTAATGATA
FRMD6 #1	122786	CAGCAAGGGTATCGACCAATT
FRMD6 #2	122786	CTAGGTTCACCTGACACTTTA
INADL #6	10207	CAGCTTCATCATACCATTCAA
INADL #10	10207	ATGCTCGTTGCTAGAGATCCA
IQGAP1 #1	8826	CTGGGAGATAATGCCCACTTA
IQGAP1 #5	8826	AAGGAGACGTCAGAACGTGGC
LATS1	9113	GAUAAAGACACUAGGAAU
LATS2	26524	CCACAGAAACCCAGCUGAATT
LIN7C #5	55327	TGGCATATTGACCCTATATAA
LIN7C #6	55327	TCAACCGTACATCAAATTATA
LLGL2 #5	3993	CAACCAGATCCTGATCGGCTA
LLGL2 #7	3993	CTGCATCTCAGTGATCCACGA
MPP5 #3	64398	AAGCCAGTTCATCATAAGGAA
MPP5 #4	64398	CGGGAGTTTCTGGATTCTTTA
MPP5 #5	64398	AACGAGGAGATCTTAACCTAT
MPP5 #6	64398	AGCATAGATTCTGTACGGCAA
NF2 #7	4771	CACCGTGAGGATCGTCACCAT
NF2 #8	4771	CCGGGTCGCGCCTGCACCGAA
PAK1 #8	5058	TCCACTGATTGCTGCAGCTAA
PAK1 #9	5058	TTGAAGAGAACTGCAACTGAA
PARD3 #3	56288	ATCGACAAATCTTATGATAAA
PARD3 #10	56288	TGGAGTAGATTTAGTGGGCAA
PARD6A #1	50855	CTCGAGGTCAATGGCATTGAA
PARD6A #5	50855	CCAGGTTTCCTCAGTCATAGA
PARD6B #7	84612	AAGGGTATCTATCAATGGTAT
PARD6B #8	84612	TAGATCCTGGTTATACGATAA
PARD6G #3	84552	AAGGTTCTCTCTGGACCGTCA
PARD6G #6	84552	CTGGGCGCGCTGCGTGATGAA
PRKCZ #5	5590	CGGAAGCATGACAGCATTAAA
PRKCZ #6	5590	GACCAAATTTACGCCATGAAA
SAV1 #4	60485	AACCATGGTTCTGAAGATTTA
SAV1 #5	60485	AACGAGTTGAGTCATCCGAAT
TJP1 #1	7082	CCGAGGCATATTTAACAGCAA
TJP1 #7	7082	CCAGTATCTGATAATGAAGAA
WWC1 #2	23286	CTGGTACAACCTTCTCAGCTA
WWC1 #3	23286	CCGCGGGACAGGTACACCAAA
Scrambled siRNA		CCGAUAUGAGUCAACAGAUTT

Table S2. Mutagenesis primers.

Application	Name	Sequence (5´-3´)

Amplification of hMPP5 for Gateway Donor vector	GW hMPP5 forward	GGGGACAAGTTTGTACAAAAAAGCAGGCTCCAC-CATGACAACATCCCATATGA
	GW hMPP5 reverse	GGGGACCACTTTGTACAAGAAA-GCTGGGTTTCACCTCAGCCAAGTGGATG
hMPP5ΔLin27 mutagenesis	hMPP5ΔLin27 forward	CAGCTAGAGCCCATTACAGAT
	hMPP5ΔLin27 reverse	ATCTTGTGCGTTGGAGATAAGA
hMPP5ΔPDZ mutagenesis	hMPP5ΔPDZ forward	CCCAGTCAACAGATCAAGC
	hMPP5ΔPDZ reverse	AGTTTCTCCTCCATACTGGC
hMPP5ΔSH3 mutagenesis	hMPP5ΔSH3 forward	AGCTTTCAGCAGCAAAGG
	hMPP5ΔSH3 reverse	GATTACTGTTTCCTTGGCAG
hMPP5ΔGuKc mutagenesis	hMPP5ΔGuKc forward	CTTGATACTGAACCTCAGTGG
	hMPP5ΔGuKc reverse	ACAGTTCTGTGGACCAATCA

Table S3. Primers for qPCR.

Gene	Acc. Number	Forward Primer (5′-3′)	Reverse primer (5′-3′)
MPP5	NM_022474.4	GCGTCAGAGGCTCATGAACAA	CTCTACCGGCTACTTCTTGGTCT
INADL (*PATJ*)	NM_176878.1	CTCCTACAGCCAGAAGATGAG	CCATAAAGCTGCATGCCATTG
LIN7C	NM_018362.4	GGAGTGGAGAAGTACCACCA	CTGATGTCCACAGTCTCATAGA

Table S4. List of primary and secondary antibodies.

Antibody (clone)	Company	Dilution	Application
**Primary Antibody**			
Actin	Santa Cruz Biotechnology	1:3,000	WB
β-Tubulin	Santa Cruz Biotechnology	1:500	WB
Ki-67 (M7240)	Dako	1:400	IHC
MCM2 (D7G11)	Cell Signaling	1:500 - 1:2,000	IHC, WB
MPP5	ProteinTech	1:100 - 1:250	IF, IHC
		1:100	PLA, IP
PARP	Cell Signaling	1:1,000	WB
TAZ	Cell Signaling	1:100 - 1:400	IF, IP, WB
TAZ	Abcam	1:50	IHC
YAP	Bethyl Laboratories	1:70	IF
YAP	Cell Signaling	1:50 - 1:400, 1:25	IHC, IP, WB, PLA
Phospho-YAP (Ser127)	Cell Signaling	1:500	WB
**Secondary Antibody**			
Cy™3 AffiniPure Donkey Anti-Rabbit	Jackson ImmunoResearch	1:500	IF
Alexa Fluor®488 AffiniPure Donkey Anti-Goat	Jackson ImmunoResearch	1:200	IF

IF: immunofluorescence, IHC: immunohistochemistry, IP: immunoprecipitation, PLA: proximity ligation assay, WB: Western immunoblotting.
